# 

**Published:** 1894-08

**Authors:** 


### CONTENTS:

### SELECTIONS.

Article I.—Mr. Tomes' Inaugural Address. - - - - 145
" II.—Nitrate of Silver as a Therapeutic Agent. - - 153
" III.—A Suggestion upon the Preparation of the Fingers and Nails for Surgical Operations. By Oscar H. Allis, M. D. - - - - - - 156
" IV.—Hypnotism. By Dr. F. O. Hetrich. - - - 158
" V.—The Immediate Root-Filling Craze. - - - 173
" VI.—Discussion on the "Retention of Artificial Dentures in Edentulous Lower Jaws." - - - - 176

### EDITORIAL, Etc.

The Dental Meetings at Old Point, Va. - - - - - 185

### OBITUARY.

Prof. R. B. Winder. - - - - - - - - 186

### MONTHLY SUMMARY.

Electricity in Dentistry. - - - - - - - 188

The Dangers of Hypnotism. - - - - - - 188

The Nails. - - - - - - - - 189

To Set a Logan Crown. - - - - - - - 190

Diagnosis of Pulp Stones. - - - - - - - 190

Silver and Aluminum. - - - - - - - 191

Hints Worth Remembering. - - - - - - 191

Protection of Cement Fillings. - - - - - - 191

Correct Articulation in Bridge-Work. - - - - - 192

Cocain Injection. - - - - - - - - 192

Alloy for Gold Solders. - - - - - - - 192

